# Distal‐type bronchiolar adenoma of the lung harboring an *EGFR exon 21 p.L858R* mutation: A case report

**DOI:** 10.1111/1759-7714.13692

**Published:** 2020-10-16

**Authors:** Chenglin Yang, Xiaoliang Wang, Jiping Da, Kai Ma

**Affiliations:** ^1^ National Cancer Center/National Clinical Research Center for Cancer/Cancer Hospital & Shenzhen Hospital Chinese Academy of Medical Sciences and Peking Union Medical College Shenzhen China

**Keywords:** Bronchiolar adenoma, ciliated muconodular papillary tumor, mutation

## Abstract

Here, we present a case of a distal‐type bronchiolar adenoma (BA) of the lung. BAs are benign lung tumors characterized by nodular proliferation of bilayered bronchiolar‐type epithelium with a continuous layer of basal cells. This patient underwent S3 segmentectomy following detection by computed tomography (CT) scan of a gradually enlarging ground‐glass nodule (GGO) over a five month period. Nodule morphology and immunophenotype were consistent with those of distal‐type BA of the lung. An epidermal growth factor receptor (*EGFR*) *exon 21 p.L858R* missense mutation was identified which, to the best of our knowledge, is the first case to be reported of a common gene mutation associated with non‐small cell lung cancer (NSCLC) being found in a BA lesion. Following surgery, the patient remains relapse‐free.

**Key points:**

**Significant findings of the study:**

- Pathological assessment of a lung nodule confirmed a papillary tumor with a double‐layered cell structure, less than typical cytoplasm, and a mixture of ciliated columnar and globular cells, consistent with a distal‐type bronchiolar adenoma.

**What this study adds:**

- This is the first report of an *EGFR exon 21 p.L858R* mutation in a bronchiolar adenoma.

## Graphical abstract

We present a case of distal‐type bronchiolar adenoma (BA) of the lung. This patient underwent S3 segmentectomy following detection by computed tomography (CT) scan of a gradually enlarging ground‐glass nodule (GGO). The morphology and immunophenotype were identical to those in distal‐type BA of the lung. An *EGFR‐21‐L858R* missense mutation was identified, which is the first case of a common gene mutation associated with non‐small cell lung cancer being found in a BA lesion.

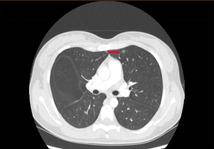

## Introduction

Bronchiolar adenomas (BAs) are defined as benign lung tumors derived from bronchiolar epithelial cells exhibiting bilayer architecture and bronchiolar differentiation.^1^ Based on morphology and immunohistochemistry, BAs are divided into proximal and distal types.^2^ Owing to the rarity of BAs, their histogenesis and molecular characteristics are unclear. Here, we report a case of distal‐type BA carrying an *EGFR exon 21 p.L858R* mutation, which is commonly associated with non‐small cell lung cancers (NSCLCs).

## Case report

**Figure 1:**
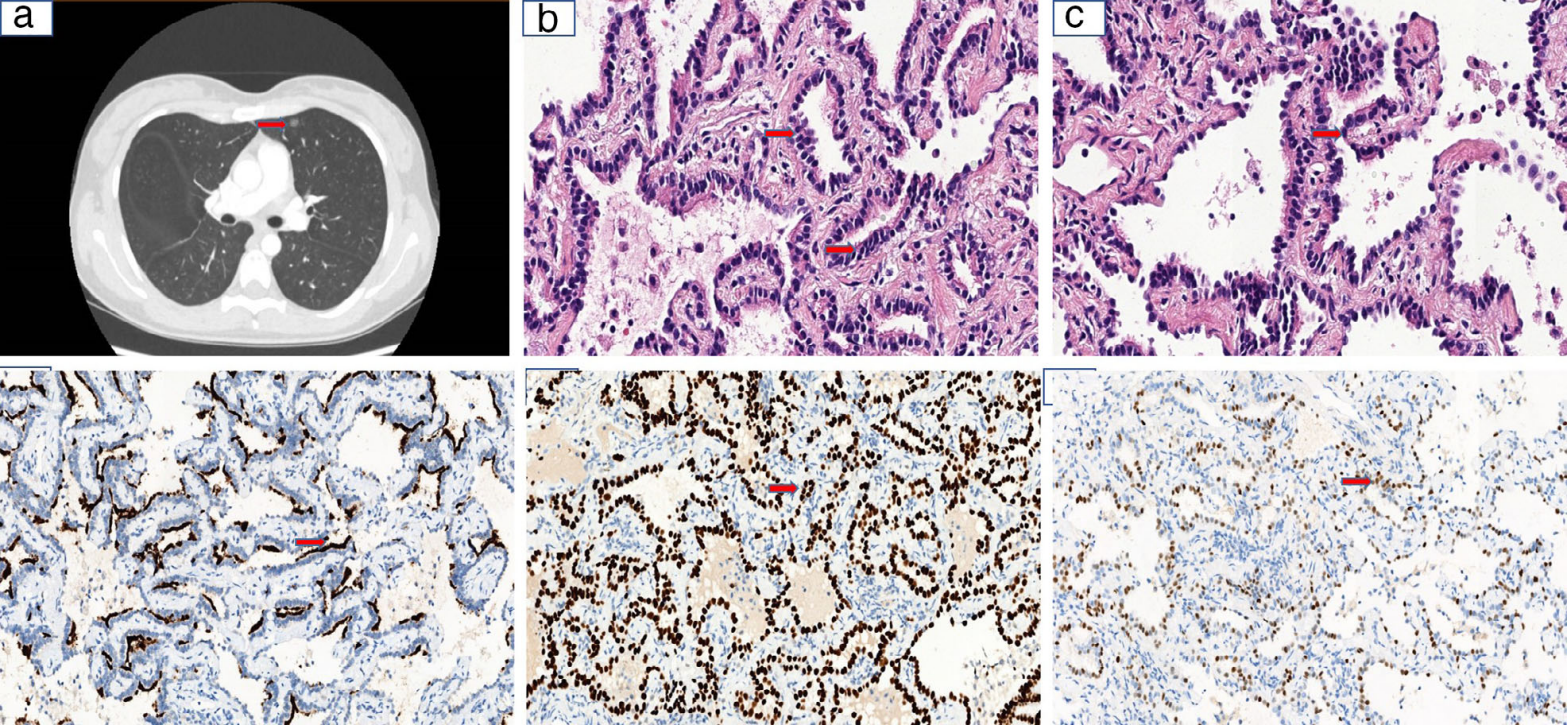
(**a**) Chest computed tomography (CT) indicated an 8 mm pure ground‐glass opacity (GGO) in the peripheral field of the left upper lobe of the lung. (**b**) Tumor cells, consisting of luminal and basal cells, proliferated along the pre‐existing alveolar septal architecture in a dual‐layer pattern (red arrow, hematoxylin‐eosin [HE] staining, ×400). (**c**) Goblet cells, basal cells, and ciliated columnar cells formed a papillary structure (red arrow, HE staining, ×400). (**d**) Positive IHC staining for thyroid transcription factor‐1. (**e** and **f**) Basal cells showed positivity for p40 and p63, respectively (red arrow, brown signals, ×400).

The patient was a 30‐year‐old Chinese woman with no relevant medical history. Five months previously, a nodule in her left lung had been detected during a regular checkup. She was a non‐smoker with no family history of malignancies. The nodule gradually increased in size from 5 to 8 mm over a period of five months. Chest computed tomography (CT) subsequently revealed a pure ground‐glass opacity (GGO) in the anterior segment of her left lung (Fig 1a). Mediastinal lymphadenopathy was absent, and serum tumor‐marker levels were normal. Early‐stage lung cancer was suspected, and she was subsequently admitted to our department for surgery. She underwent an S3 segmentectomy via uniportal video‐assisted thoracoscopic surgery (VATS). The intraoperative frozen section histology confirmed that it was a distal‐type BA. Nine months post‐surgery, the patient remains relapse‐free.

Histological examination revealed a dual‐layered architecture with discontinuous areas (Fig 1b). The tumor cells, comprising luminal and basal cells, were cuboidal, with small round or oval nuclei, less than typical cytoplasm, and papillary and lepidic structures. Nuclear atypia, mitosis, and necrosis were absent (Fig 1c). Immunohistochemistry revealed that most tumor cells were positive for thyroid transcription factor‐1(TTF‐1) (Fig 1d) and p40. Basaloid cells were identified based on p63 positivity (Fig 1e–f); some basaloid cells showed lepidic growth patterns. Malignancy could not be ruled out due to discontinuity in the double‐layered structure and lepidic growth of basal cells. The ciliated structures present in the luminal cells distinguished this tumor from invasive adenocarcinoma. The morphology and immunophenotype were consistent with those of distal‐type BAs of the lung.

The sample was tested in a clinical genomic testing facility (Nanjing Geneseeq Technology Inc., Nanjing, China) with protocols approved by the ethics committee of Shenzhen Cancer Hospital. Mutation analysis of *EGFR* was performed using next‐generation sequencing (NGS). An *EGFR exon 21 p.L858R* missense mutation was identified.

## Discussion

Lung BAs^2^ have been recently defined as rare peripheral lung tumors with nodular proliferation of bilateral benign bronchiolar epithelium with a continuous basal cell layer. BAs were first described as ciliated muconodular papillary tumors (CMPTs) in 2002.^3^ CMPTs are characterized by tripartite cellular components consisting of ciliated columnar cells, mucosal cells, and basal cells with a predominant papillary architecture. Most BA lesions do not meet all the CMPT diagnostic criteria; BAs usually only manifest as focal or nonpapillary structures which contain a variable number of ciliated cells and mucous cells; however, some lesions lack one or both of these components. Morphological and immunohistochemical characteristics are used to differentiate proximal‐type BAs from distal‐type BAs based on similarities to the proximal or distal respiratory bronchioles, respectively. In our case, a double‐layered structure was apparent with no visible mucosal structures; most of the luminal cells had cilia, and the morphology and immunophenotype were identical to those of distal‐type BA of the lung.

Most BA cases occur in patients aged >60 years (median age: 67 years).^4^ This rare tumor is mainly seen in East Asia and less frequently in western countries.^5^ More than 80% of BAs are found in the lower lobes of the lungs and lung periphery adjacent to the pleura. On imaging, most nodules appear solid or partially solid, with a few exhibiting ground‐glass opacity (GGO) features.^4^ Most BAs are discovered incidentally during physical examination, and follow‐up after several months shows that some lesions have enlarged.^6^, ^7^, ^8^, ^9^, ^10^ In this case, follow‐up with CT imaging at six months revealed a 3 mm lesion increase, suggesting the lesion was of a neoplastic nature. Because diagnostic imaging indicated early‐stage lung cancer, the patient required surgical treatment, but refused CT‐guided fine‐needle aspiration before surgery. Although the malignant potential of BAs remains controversial, BAs are inert tumors with good prognoses. Most BA patients undergo partial resection and achieve long‐term recurrence‐free survival^4^; and excessive resection and lymph node removal are unnecessary.

The high incidence of driver gene mutations in reported cases suggests that BA is a neoplastic lesion; whether it is an early lesion associated with lung cancer remains controversial. Serine‐threonine protein kinase B‐RAF (*BRAF*) mutations are more common in distal‐type lesions compared with proximal lesions (54% vs. 13%, respectively).^2^ However, our patient did not carry a *BRAF* mutation. This is the first reported case involving *EGFR exon 21 p.L858R*, a gene mutation associated with non‐small cell lung cancer (NSCLC), in a BA. Further studies are needed to investigate the malignant potential of BAs and their progression to adenocarcinoma.

## Disclosure

The authors declare no competing interests.
